# Understanding and Targeting HBV Transcription and Post-Transcriptional Regulation

**DOI:** 10.3390/v16111793

**Published:** 2024-11-19

**Authors:** Simon P. Fletcher, Rudolf K. Beran

**Affiliations:** Gilead Sciences, Foster City, CA 94404, USA

Chronic hepatitis B (CHB) affects approximately 300 million people worldwide and current therapies rarely cure it. One potential strategy for achieving a functional HBV cure is to permanently silence expression from the HBV genome, covalently closed circular DNA (cccDNA). There has thus been a push in the field to better understand HBV transcription and post-transcriptional regulation. In recent years, we have learned that the host Smc5/6 complex binds cccDNA, likely within PML nuclear bodies [1,2], when cccDNA is supercoiled and actively transcribing to silence its transcription [3,4]. Various other host factors, such as HNF1a and HNF4a, have been shown to enhance HBV transcription, while CTCF binds HBV Enhancer I to repress transcription. In addition, cccDNA transcription can be epigenetically modulated in either a positive or negative manner. For example, HDAC11 deacetylates cccDNA, thereby repressing cccDNA transcription, whereas HAT1 acetylates cccDNA and promotes viral gene expression [5]. Furthermore, the post-transcriptional regulatory element of HBV is known to play a role in controlling the stability and nuclear export of HBV RNA [6].

Understanding HBV transcription and post-transcriptional regulation has helped identify new therapies able to silence cccDNA. For example, the cellular factors PAPD5 and PAPD7 were identified as being required for HBV RNA stabilization and as therapeutic targets for HBV cure [7]. Furthermore, characterizing HBV transcription from cccDNA as well as from HBV integrants was critical for understanding how to design siRNAs and antisense oligonucleotides (ASOs) that target HBV RNAs from both sources [8]. 

Antivirals that promote HBV RNA degradation, such as HBV siRNAs and ASOs, and small molecules that block HBV RNA polyadenylation continue to be investigated. HBV siRNAs targeting the HBx transcript reduce the levels of all HBV RNAs, as well as HBsAg levels, but siRNAs are unable to achieve a functional cure in most cases [9]. Bepirovirsen is an ASO that targets HBV RNA, but it also has undefined immunomodulatory effects. However, even with Bepirovirsen’s dual mechanism of action, its response rate, defined as HBsAg < 0.05 IU/mL, was only observed as being 9–10% of patients 24 weeks post-treatment and primarily occurred in patients who started out with low HBsAg levels [10]. Thus, permanently silencing HBV cccDNA expression through transcriptional or post-transcriptional inhibition remains elusive.

In this Special Issue of *Viruses*, we have put together a wide range of papers and reviews concerning topics relevant to the transcription and post-transcriptional regulation of HBV. These include papers on innate immune factors that reduce HBV RNA levels, a small molecule that destabilizes HBV RNA, the HBV post-transcriptional regulatory element which controls HBV RNA stability and nuclear export, and a review of what is known about cccDNA formation and regulation.
